# Supplementary material for: Effects of ankle Kinesio taping on knee and ankle joint biomechanics during unanticipated jumps in collegiate athletes
Source: PLoS One. 2024 Aug 1;19(8):e0305480. doi: 10.1371/journal.pone.0305480 (PMC11293634; doi:10.1371/journal.pone.0305480)
Supplement: Attachment — Submitted filename: Response to reviewers.docx [file pone.0305480.s002.docx]

**Summary of a Point-by-Point Response to Reviewers’ Critiques**

Manuscript PONE-D-24-02413 entitled “Effects of Ankle Kinesio Taping on Knee and Ankle Joint Biomechanics During Unanticipated Jumps in Collegiate Athletes”

We sincerely thank the editor and all reviewers for the kind consideration and constructive comments on our manuscript. Due to your valuable suggestions, the entire manuscript will be further refined. All of us authors have carefully read the comments that you have given us, and have discussed and revised each of these issues. Revised portions are marked yellow in the manuscript. The main corrections in the manuscript and the responses to the reviewers' comments are listed below.

Best regards

**Reviewer #1:**

*The manuscript entitled "Effects of Ankle Kinesio Taping on Knee and Ankle Joint Biomechanics During Unanticipated Jumps in Collegiate Athletes", aimed to investigate the effects of ankle KT application on the kinematic and kinetic characteristics of the knee and ankle joints during unanticipated jump tasks. The authors have conducted motion capture analyses to 18 participants and concluded that KT reduced peak ankle inversion angle, peak vertical ground reaction force, and increased peak knee flexion. While the paper addresses an interesting issue, it is not publishable in its current form. There are several critical concerns about the experiment design that greatly undermine the significance of the research outcomes. In addition, the language quality of the manuscript falls short of publication standards. Many sentences in the content are confusing and several arguments are self-contradictory.*

**Reply:** Thank you very much for your feedback and suggestions. We sincerely apologize for the issues in the manuscript. Regarding the concerns you raised about the experimental design, we have addressed them by adding details about the unanticipated conditions in the manuscript.

Line 128-129：The signal light activated immediately (within 1 s) following the beginning of the participants' running.

On the one hand, participants were unaware of the direction of the jump before commencing the run. Within 1 s of initiating the run, they were informed of the jump direction through a signal light. Consequently, they reacted and completed the jumping task without prior preparation, providing them with short time for motor adjustment. On the other hand, this parallels unanticipated movements observed in actual sports. Such as basketball players react to stimuli while running and subsequently execute jumping movements. We sincerely apologize for the language issue of the manuscript you mentioned. The language and grammar of this manuscript have been polished by a native English speaker familiar with medical writing.

**General comments**

*1. Based on the authors’ introduction, “unanticipated movement tasks” appeared to be an important element of the study and served as a key factor that would preassembly influence the research outcome. However, it is very the participants were considered situated in an “unanticipated” scenario using the current research setup as described in the manuscript. I believe that participants were well aware of the jumping tasks before commencement and they were only required to respond to three alternative conditions. Signals were given to the participants before they kicked off while they had the extra time to prepare for the correct tasks, particularly landing, during running on a 2-meter runway. I wonder how much the “unanticipated” element was included in the research outcome.*

**Reply:** Thank you very much for your valuable comments on our study. We are very sorry for the misunderstanding. In fact, the subjects did not know the direction of the jump until they started running. A trained experimenter was responsible for sending the signal, which turned on the light within 1 second after the subject started running. Therefore, they react and complete jumping movements without preparation. We have added settings regarding unexpected conditions in the manuscript.

Line 128-129：The signal light activated immediately (within 1 s) following the beginning of the participants' running.

*2. I think the effects of KT on ankle kinematics are well established, that is why it is widely applied in sports and clinics. Without involving other factors in the research setup, the study outcomes added very limited knowledge to the current understanding of the mechanism.*

**Reply:** Thank you very much for your valuable comments on our study. On the one hand, current studies exploring KT's effect on the biomechanical characteristics of participants have some conflicting conclusions. On the other hand, most studies selected test movements as expected movements, while this study used unanticipated movements for testing. This testing method is closer to the way ankle sprains occur in sports activities, and therefore is more representative of the actual effect of KT.

Specific comments

*Line 25. I think it should be “anticipated movements” instead of “unanticipated movements”*

**Reply:** Thank you very much for your valuable comments on our study. We are very sorry for the mistake. We have revised it.

Line 25：Most biomechanical research on the application of Kinesio taping (KT) to the ankle joint focused on testing anticipated movements

*Line 54-55. “reduced limitations in ankle dorsiflexion range of motion.” What does that mean? Is not KT supposed to constrain ankle motion?*

**Reply:** Thank you very much for your valuable comments on our study. Chang et al, showed that compared with athletic taping, KT has less restriction on ankle dorsiflexion movement. We are very sorry for the misunderstanding. We have revised it.

Line 55-56: Chang et al. [6] found that KT could improve balance function in healthy collegiate athletes and did not restrict ankle dorsiflexion range of motion.

*Line 65. Based on the context, it should be “suboptimal position” instead of “optimal position”*

**Reply:** Thank you very much for your valuable comments on our study. We are very sorry for the misunderstanding. We have revised it.

Line 65-66: This alteration may increase the risk of ankle sprains by preventing the body from promptly adjusting its movements and absorbing the impact of landing [13].

*Line 81-82. There is insufficient discussion in the introduction to support the authors’ hypotheses. In other words, why did the author hypothesize that KT would reduce peak ankle plantarflexion and inversion angles and moments, and increase peak knee flexion angle?*

**Reply:** Thank you very much for your valuable comments on our study. We have deleted the research hypotheses in the introduction.

*Line 91. How do you define “engaged”?*

**Reply:** Thank you very much for your valuable comments on our study. We are very sorry for the misunderstanding. We recruit athletes majoring in basketball, football or volleyball at Wuhan Sports University (Tegner score ≥7). They participated in sports ≥3 times a week, each session lasting ≥45 minutes.

*Line 110. Based on Figure 1, I think it should be “lateral malleolus.”*

**Reply:** Thank you very much for your valuable comments on our study. We have not introduced the peroneus longus muscle taping method in detail before, which has caused misunderstandings, and we are very sorry for this. We have modified the description of this section.

Line 108-110: The anchor was attached to the fibular head, and the tail was attached to the medial malleolus. The taping covered the peroneus longus muscle belly, bypassing the sole of the foot to the medial malleolus.

*Line 135. The speed value (2.7 ± 0.4 m/s) looks like a actual outcome rather than a target value.*

**Reply:** Thank you very much for your valuable comments on our study. In order to reduce the heterogeneity of the study and reduce the differences in the study results caused by the different running speeds of the subjects. We limited the running speed of the subjects. Before the formal test, the subjects performed running exercises so that they could maintain a speed of (2.7 ± 0.4 m/s) during the formal test. And we have excluded data that does not fit this speed range.

*Line 138. I would not call it “blinded.” The participants were very well aware of the tasks after signals were given.*

**Reply:** Thank you very much for your valuable comments on our study. We are very sorry for the misunderstanding. In fact, the subjects did not know the direction of the jump until they started running. A trained experimenter was responsible for sending the signal, which turned on the light within 1 second after the subject started running. Therefore, they react and complete jumping movements without preparation. We have added settings for unexpected conditions in the article.

Line 128-129：The signal light activated immediately (within 1 s) following the beginning of the participants' running.

*Line 156-157. Incorrect punctuation.*

**Reply:** Thank you very much for your valuable comments on our study. We are very sorry for the mistake. We have revised it.

Line 157: and time to peak vertical ground reaction force (vGRF).

*Line 198-199. How is the KT method applied in the study able to “reduce gastrocnemius contraction” while also “promote tibialis anterior contraction”?*

**Reply:** We performed inhibitory taping on the gastrocnemius muscle (Fig 1C) and facilitative taping on the tibialis anterior muscle (Fig 1C). The facilitative taping method refers to applying KT from the starting point to the ending point of the muscle fiber. This will cause the muscle ending point to retract in the direction of the starting point, improve contraction, and thereby increase muscle strength. This change in muscle length may optimize the muscle's length-tension relationship, thereby improving the muscle's force-producing capacity. The inhibitory taping method refers to the application of KT from the insertion point to the starting point of the muscle fiber, which can inhibit muscle function (DOI: 10.2340/16501977-2845). The tibialis anterior and gastrocnemius are a pair of muscles that are antagonistic to each other. We use this taping method to reduce the ankle plantar flexion angle.

*Line 216-217. Again, it should be “lateral malleolus.”*

**Reply:** Thank you very much for your valuable comments on our study. We have not introduced the peroneus longus muscle taping method in detail before, which has caused misunderstandings, and we are very sorry for this. We have modified the description of this section.

Line 108-110: The anchor was attached to the fibular head, and the tail was attached to the medial malleolus. The taping covered the peroneus longus muscle belly, bypassing the sole of the foot to the medial malleolus.

*Line 219. “Poorer function” in what aspect?*

**Reply:** Thank you very much for your valuable comments on our study. We have made revisions and detailed explanations in the manuscript.

Line 221-229: Additionally, it may be attributed to the improvement of proprioception by KT, enabling the participants to accurately perceive joint angles, thereby exhibiting reduced ankle inversion angle[5]. Wei et al.[37] also indicated that patients with poor proprioception may be more sensitive to KT, more susceptible to its facilitative effects, and transmit more proprioceptive information from joint structures to the nervous system. As a result, patients with poor proprioception tended to receive more benefits than healthy participants with good proprioception. Although the participants in the present study were healthy athletes, the test movements selected in our study were unanticipated jumps. Considering the lower stability and poorer proprioceptive input of athletes during unanticipated jumps, KT may be more effective in promoting proprioception.

*Line 229. The authors emphasized many times that lower limb joints were kinematically coupled and the knee joint would supplement restricted ankle motion under intervention conditions. Then here comes a contradictory statement of “Given that the taping did not cover the knee joint, the likelihood of KT improving peak knee flexion angle through mechanical effects is relatively low.”*

**Reply:** Thank you very much for your valuable comments on our study. We mentioned in the introduction that the lower limb joint is a whole, and changes in the biomechanical characteristics of the ankle joint will affect the knee joint. Since KT does not directly cover the knee joint, we believe that the probability of KT changing the biomechanical characteristics of the knee joint through mechanical effects is small. Therefore, it may be that KT changes the biomechanical characteristics of the ankle joint, thereby indirectly affecting the biomechanical characteristics of the knee joint. We are very sorry for the mistake. We have revised it.

Line 233-235: The result may be attributed to the alteration of ankle joint biomechanical characteristics by KT, thereby indirectly influencing knee joint biomechanical features.

*Line 244. The logic of “limiting a portion of joint ROM while maintaining joint stability [41]” is ambiguous. Suggest to rephrase it.*

Reply: Thank you very much for your valuable comments on our study. We are very sorry for the mistake. We have revised it.

Line 247-249: Yalfani et al. [40] adopted a KT application method similar to the basket weaves technique of athletic taping. This approach will mechanically restrict the foot and ankle, thereby reducing the range of motion of the lower limb joints [41].

*Line 253-254. How does increased knee flexion “reducing the risk of ACL injuries?”*

**Reply:** Thank you very much for your valuable comments on our study. Some studies have shown that reduced knee flexion angle and increased vGRF peak are related factors of ACL injury. The results of this study show that KT can increase knee flexion angle and reduce vGRF peak, suggesting that KT may have a potential role in reducing the risk of ACL. We do not assert here that KT reduces the risk of ACL injury. However, we believe that the results of this study have implications for future research.

*Line 259. “In addition, the taping retraction from the medial malleolus to the lateral malleolus imparts tension to ankle eversion.” There were no tapes applied in this manner in the study.*

**Reply:** Thank you very much for your valuable comments on our study. We are very sorry for the misunderstanding. The description in the article is correct. We have added instructions for peroneus longus taping methods to the article.

Line 108-110: The anchor was attached to the fibular head, and the tail was attached to the medial malleolus. The taping covered the peroneus longus muscle belly, bypassing the sole of the foot to the medial malleolus.

*Line 267. What is CAI?*

**Reply:** Thank you very much for your valuable comments on our study. We are very sorry for the mistake. We have revised it.

Line 271-273: A study indicated that patients with chronic ankle instability exhibit different biomechanical patterns and muscle activation under unexpected conditions compared to those under expected conditions [43].

*All points made in the Limitations sections were off-point. EMG was not the research focus and therefore not supposed to be the limitation. KT is usually not expected to provide sustainable and long-term effects so studying “immediate effects” falls short of being one of the limitations. Non-dominant side also has its own “habitual patterns.” The arguments here are misleading.*

**Reply:** Thank you very much for your valuable comments on our study. We revised and supplemented the study limitations.

Line 283-288: First, only healthy individuals without lower limb injuries included in our study. Therefore, the results of our study may not be generalized to individuals with pathology. However, studies in healthy participants may have implications for the prevention of sports injuries. Another limitation is that men and women were not analyzed separately because of the relatively small sample size, despite some evidence of biomechanical differences between sexes.

Reviewer #2:

*The article contains a novel and applicable criteria and sounds very good.*

*Introduction meets the general structure and reflects the importance of the research.*

*Sampling methods and methodology also are well prepared and well orianted justifications regarding the findings are available.*

**Reply:** We appreciate your summary of the manuscript and encouraging comment. We have modified the article in the hope that it can provide a basis for the use of KT in actual sports and provide valuable information and inspiration for subsequent research.
