# Supplementary material for: Effects of ankle Kinesio taping on knee and ankle joint biomechanics during unanticipated jumps in collegiate athletes
Source: PLoS One. 2024 Aug 1;19(8):e0305480. doi: 10.1371/journal.pone.0305480 (PMC11293634; doi:10.1371/journal.pone.0305480)
Supplement: Attachment — Submitted filename: Response to reviewers.docx [file pone.0305480.s003.docx]

**Summary of a Point-by-Point Response to Reviewers’ Critiques**

Manuscript PONE-D-24-02413R1 entitled “Effects of Ankle Kinesio Taping on Knee and Ankle Joint Biomechanics During Unanticipated Jumps in Collegiate Athletes”

Thank you very much for your kindly comments on our manuscript. There is no doubt that these comments are valuable and very helpful for revising and improving our manuscript. In what follows, we would like to answer the questions you mentioned and give detailed account of the changes made to the manuscript. Revised portions are marked yellow in the manuscript. The main corrections in the manuscript and the responses to the reviewers' comments are listed below.

**Reviewer #1:**

The authors have addressed all my previous comments properly and sufficiently. The manuscript in its current form has advanced to a status that can be considered for publication, on the condition that the following follow-up questions can be

*1. Further to my comments to Line 198-199 in the first-round revision:*

*Based on the citations provided by the authors as well as the relevant research findings in the literature, a taping method is considered to inhibit muscle contraction only when the tapes are applied with tension across the muscle belly, namely perpendicular to the direction of the muscle fibers. According to the authors’ depictions and the figure’s manifestation, the Kinesio tapes were applied in Y shape along the belly of the two gastronomic heads in the current study, which I hardly agree would impose inhibitory effects on muscle contraction.*

*I suggest revising relevant content to refrain from emphasizing the “inhibitory effects” of the taps applied on gastrocnemius.*

**Reply:** Thank you for pointing this out. We have removed the reference to gastrocnemius inhibition. The previous content is in lines 200-201.

*2. Further to my comments to Line 253-24 and Line 283-288.*

*Please attach references to support the corresponding arguments, for example, increasing knee flexion reduces the risk of ACL injury and evidence of gender differences in knee/ankle biomechanics during unanticipated jumping.*

**Reply:** Thanks for your valuable counsel. We have added references to knee flexion angle(doi: 10.2519/jospt.2015.5785. doi: 10.1589/jpts.28.2316. Epub 2016 Aug 31.) and gender differences(doi: 10.1080/02640414.2020.1837481. doi: 10.1136/bjsm.2008.055954.). In addition, we performed subgroup analyses of all outcome indicators according to gender differences, and the results support the statement in limitations(**Table 3**). The results of subgroup analysis of gender differences are in the supporting information, and the file name is Two-way repeated measures ANOVA.

Line 255-257: This can not only decrease the load on lateral ankle ligaments and mitigate the risk of ankle sprains but also may have potential implications for reducing the risk of ACL injuries[42, 43].

Line 285-287: Another limitation is that men and women were not analyzed separately because of the relatively small sample size, despite some evidence of biomechanical differences between sexes[46, 47].

**Table 3. Kinetic characteristics of left jumps**

| Outcome | Male | | | Female | | | Taping effect | Sex effect | Interaction effect |
| --- | --- | --- | --- | --- | --- | --- | --- | --- | --- |
|  | NT | PT | KT | NT | PT | KT |  |  |  |
| Peak  vGRF(BW) | 1.94±0.12 | 1.99±0.24 | 1.95±0.21 | 1.85±0.20 | 1.79±0.09 | 1.82±0.10 | p=0.983 | p=0.069 | p=0.392 |
| Peak knee flexion moment (Nm/kg) | 0.35±0.10 | 0.37±0.18 | 0.37±0.16 | 0.62±0.21 | 0.61±0.31 | 0.48±0.30 | p=0.325 | **p=0.024^*^** | p=0.216 |
| Peak knee valgus moment (Nm/kg) | 0.16±0.08 | 0.18±0.10 | 0.18±0.12 | 0.20±0.12 | 0.20±0.11 | 0.20±0.11 | p=0.507 | p=0.610 | p=0.745 |
| Peak knee internal rotation moment (Nm/kg) | 0.08±0.04 | 0.07±0.02 | 0.07±0.04 | 0.10±0.03 | 0.09±0.06 | 0.09±0.06 | p=0.575 | p=0.276 | p=0.984 |
| Peak ankle plantarflexion moment (Nm/kg) | 1.39±0.25 | 1.47±0.30 | 1.38±0.19 | 1.47±0.34 | 1.41±0.33 | 1.42±0.24 | p=0.805 | p=0.845 | p=0.546 |
| Peak ankle inversion moment (Nm/kg) | 0.81±0.19 | 0.77±0.15 | 0.64±0.15 | 0.81±0.22 | 0.78±0.15 | 0.70±0.23 | **P<0.001^*^** | p=0.718 | p=0.654 |

Note: ^*^significant difference

**Reviewer #2:**

In this manuscript, the authors investigated the effects of ankle kinesio taping on the kinematic and kinetics of knee and ankle joints during jump tasks in athletes.

*1. Lines 46-47: Please update the numbers to more recent references as the currently cited references are 40 and 7 years old, respectively..*

**Reply:** Thanks for your suggestion. We have updated the references and made corresponding revisions in the manuscript(doi: 10.1136/bmjopen-2022-069867. doi: 10.1016/j.jshs.2019.07.009.).

Lines 46-48: Globally, approximately 1 in 10,000 people suffer ankle sprains daily, with 74% of these cases eventually progressing to chronic ankle instability, which poses a substantial healthcare burden[1, 2].

*2. Lines 74-78: These sentences are confusing and should be removed since the present study did not evaluate the ACL property.*

**Reply:** Thanks for your reminding. Numerous studies have indicated a correlation between ankle sprains and ACL injuries. As the knee joint is an adjacent joint to the ankle joint, the alterations of ankle biomechanical characteristics can induce corresponding changes in knee biomechanical characteristics. The biomechanical indicators of the knee joint used in this study have also been demonstrated to be associated with the risk of ACL injuries, such as knee valgus angle. Therefore, this study elucidated the risk of ACL injury. As you pointed out, since we did not directly assess ACL attributes, we have downplayed the content related to ACL injuries in the discussion and conclusion.

*3. The objective of the study should be rewrite and included in a separate paragraph at the end of introduction section. Please conclude with a brief statement of the overall aim of the work and a comment about whether that aim was achieved.*

**Reply:** Thanks for your valuable counsel. We have rewritten the final paragraph of the introduction according to your suggestion. Additionally, we briefly outline whether this study achieved the expected results in the first paragraph of the discussion.

Line 79-84: The present study employed a randomized signal light system emitting three distinct signals to simulate an unanticipated environment similar to actual movement. We compared the differences in knee and ankle biomechanical characteristics among collegiate athletes under three treatments: no taping (NT), placebo taping (PT), and KT, thus providing a reference for the application of KT in actual sports. The hypothesis of this study was that KT can reduce peak ankle plantarflexion and inversion angles and moments, and increase peak knee flexion angle.

Line 193-195: Partially consistent with the expected results, the present study revealed that ankle KT reduced peak ankle inversion angle, peak vGRF, and peak ankle inversion moment and increased peak knee flexion angle.

*4. Please rewrite and reorganize the last paragraph of the Introduction section.*

**Reply:** Thanks for your valuable counsel. We have rewritten the final paragraph of the introduction according to your suggestion.

Line 79-84: The present study employed a randomized signal light system emitting three distinct signals to simulate an unanticipated environment similar to actual movement. We compared the differences in knee and ankle biomechanical characteristics among collegiate athletes under three treatments: no taping (NT), placebo taping (PT), and KT, thus providing a reference for the application of KT in actual sports. The hypothesis of this study was that KT can reduce peak ankle plantarflexion and inversion angles and moments, and increase peak knee flexion angle.

*5. Only 18 subjects were included. The sample size is too small, making the results less reliable when extrapolating to a larger population. While G-Power was used to calculate the sample size, it is assumed that gender does not have an impact on the results. However, if gender does indeed influence the outcomes, the current sample size would be considered inadequate. A larger sample size should be considered to enhance the robustness of the results.*

**Reply:** We apologize for this confusion. Due to the limited sample size, we did not analyze male and female participants separately. Therefore, we have accounted for the limitations with the intention of providing some valuable information for future research. We recommend that future research should utilize a larger sample size and conduct analyses based on gender. Moreover, we employed two-way repeated measures ANOVA to recalculate all outcome indicators. The results indicated that there are indeed gender differences in the biomechanical characteristics of participants during unanticipated jumps(**Table 3**). The results of subgroup analysis of gender differences are in the supporting information, and the file name is Two-way repeated measures ANOVA.

**Table 3. Kinetic characteristics of left jumps**

| Outcome | Male | | | Female | | | Taping effect | Sex effect | Interaction effect |
| --- | --- | --- | --- | --- | --- | --- | --- | --- | --- |
|  | NT | PT | KT | NT | PT | KT |  |  |  |
| Peak  vGRF(BW) | 1.94±0.12 | 1.99±0.24 | 1.95±0.21 | 1.85±0.20 | 1.79±0.09 | 1.82±0.10 | p=0.983 | p=0.069 | p=0.392 |
| Peak knee flexion moment (Nm/kg) | 0.35±0.10 | 0.37±0.18 | 0.37±0.16 | 0.62±0.21 | 0.61±0.31 | 0.48±0.30 | p=0.325 | **p=0.024^*^** | p=0.216 |
| Peak knee valgus moment (Nm/kg) | 0.16±0.08 | 0.18±0.10 | 0.18±0.12 | 0.20±0.12 | 0.20±0.11 | 0.20±0.11 | p=0.507 | p=0.610 | p=0.745 |
| Peak knee internal rotation moment (Nm/kg) | 0.08±0.04 | 0.07±0.02 | 0.07±0.04 | 0.10±0.03 | 0.09±0.06 | 0.09±0.06 | p=0.575 | p=0.276 | p=0.984 |
| Peak ankle plantarflexion moment (Nm/kg) | 1.39±0.25 | 1.47±0.30 | 1.38±0.19 | 1.47±0.34 | 1.41±0.33 | 1.42±0.24 | p=0.805 | p=0.845 | p=0.546 |
| Peak ankle inversion moment (Nm/kg) | 0.81±0.19 | 0.77±0.15 | 0.64±0.15 | 0.81±0.22 | 0.78±0.15 | 0.70±0.23 | **P<0.001^*^** | p=0.718 | p=0.654 |

Note: ^*^significant difference

*6. Furthermore, it is crucial to investigate potential gender effects and whether the same results can be expected across different gender groups. Including an analysis of gender effects is important to ensure a comprehensive understanding of the outcomes. Additionally, it is assumed that gender does not have an impact on the results in the manuscript. However, if gender does indeed influence the outcomes, the current sample size would be considered inadequate.*

**Reply:** Your suggestion is highly valuable. We also believe it is important to analyze biomechanical characteristics during unanticipated jumps based on gender differences. However, the primary aim of this study was to investigate knee and ankle joint biomechanical characteristics during unanticipated jumps. Therefore, we have accounted for the limitations with the intention of providing some valuable information for future research. We recommend that future research should utilize a larger sample size and conduct analyses based on gender. Moreover, we employed two-way repeated measures ANOVA to recalculate all outcome indicators. The results indicated that there are indeed gender differences in the biomechanical characteristics of participants during unanticipated jumps(**Table 3**). The results of subgroup analysis of gender differences are in the supporting information, and the file name is Two-way repeated measures ANOVA.

**Table 3. Kinetic characteristics of left jumps**

| Outcome | Male | | | Female | | | Taping effect | Sex effect | Interaction effect |
| --- | --- | --- | --- | --- | --- | --- | --- | --- | --- |
|  | NT | PT | KT | NT | PT | KT |  |  |  |
| Peak  vGRF(BW) | 1.94±0.12 | 1.99±0.24 | 1.95±0.21 | 1.85±0.20 | 1.79±0.09 | 1.82±0.10 | p=0.983 | p=0.069 | p=0.392 |
| Peak knee flexion moment (Nm/kg) | 0.35±0.10 | 0.37±0.18 | 0.37±0.16 | 0.62±0.21 | 0.61±0.31 | 0.48±0.30 | p=0.325 | **p=0.024^*^** | p=0.216 |
| Peak knee valgus moment (Nm/kg) | 0.16±0.08 | 0.18±0.10 | 0.18±0.12 | 0.20±0.12 | 0.20±0.11 | 0.20±0.11 | p=0.507 | p=0.610 | p=0.745 |
| Peak knee internal rotation moment (Nm/kg) | 0.08±0.04 | 0.07±0.02 | 0.07±0.04 | 0.10±0.03 | 0.09±0.06 | 0.09±0.06 | p=0.575 | p=0.276 | p=0.984 |
| Peak ankle plantarflexion moment (Nm/kg) | 1.39±0.25 | 1.47±0.30 | 1.38±0.19 | 1.47±0.34 | 1.41±0.33 | 1.42±0.24 | p=0.805 | p=0.845 | p=0.546 |
| Peak ankle inversion moment (Nm/kg) | 0.81±0.19 | 0.77±0.15 | 0.64±0.15 | 0.81±0.22 | 0.78±0.15 | 0.70±0.23 | **P<0.001^*^** | p=0.718 | p=0.654 |

Note: ^*^significant difference

*7. Figure 1: Please include a figure illustrating the three treatments: NT, PT, and KT, to ensure clarity and facilitate understanding.*

**Reply:** Thanks for your suggestion. We have replaced the figure 1, and the replaced image can clearly reflect the processing methods under NT, PT, and KT conditions. We have also provided specific instructions in the taping procedure for the three conditions.

**Fig 1. Interventions under three conditions. (A) Kinesio taping, (B) placebo taping, (C) no taping**

*8. Figure 2: Please include a figure illustrating the three different jump patterns to provide visual clarity and improve understanding of the experimental design.*

**Reply:** Thank you for your suggestion. Since only left and right jumping maneuvers were analyzed in this study, we provided jump pattern diagrams illustrating the jumping methods for both left and right jumps.

**Fig 2. Unanticipated single-leg jump technique. (A) Left jump, (B) Right jump; Solid arrows denote the direction of movement**

*9. Please clarify whether all participants performed all three different jump patterns using their right leg. Please include the information in the data collection section.*

**Reply:** We apologize for the confusion. We have revised the description of the jump patterns accordingly. When participants performed left and right jumps, their right leg landed on the force plate. But for double-leg jumping, both legs landed separately on two force plates.

Line 131-135: When the signal light turned blue, the participants ran and landed on the right force plate with the right leg and quickly jumped to the left force plate. When it turned green, they ran and landed on the left force plate with the right leg and then jumped to the right force plate. When the light was red, the participants ran, landed each leg on two separate plates, and performed a vertical jump **(Fig 2)**

*10. Conclusion section should be improved. The authors should provide a concise summary of the main findings and their significance, as well as potential directions for future research.*

**Reply:** Your suggestion is highly valuable, and we have made the requested revisions accordingly.

Line 289-293: During unanticipated jump maneuvers, KT reduced peak ankle inversion angle, peak vGRF, and peak ankle inversion moment and increased peak knee flexion angle in collegiate athletes. This suggests that KT may have a positive effect on reducing the risk of ankle sprains in healthy individuals. Future research should explore whether KT can also mitigate the risk of ankle sprains in individuals with musculoskeletal disorders under unanticipated conditions.
